# The novel Y371D myocilin mutation causes an aggressive form of juvenile open-angle glaucoma in a Caucasian family from the Middle-East

**Published:** 2009-09-24

**Authors:** Inbal Avisar, Moshe Lusky, Anat Robinson, Mordechai Shohat, Stéphane Dubois, Vincent Raymond, Dan D. Gaton

**Affiliations:** 1 Department of Ophthalmology, Rabin Medical Center, Beilinson Campus, Petah Tiqwa, Israel; 2 Recanati Institute for Medical Genetics, Rabin Medical Center and FMRC; both affiliated with Sackler Faculty of Medicine, Tel Aviv University, Tel Aviv, Israel; 3 Laboratory of Ocular Genetics and Genomics, CREMOG, CHUL (Laval University Medical Center) Research Center, Québec City, Canada

## Abstract

**Purpose:**

To search for the genetic cause of juvenile open-angle glaucoma (JOAG) in a Caucasian family and to perform genotype/phenotype correlation studies in the kindred.

**Methods:**

Six members of a three-generation family originating from Uzbekistan and now living in the Middle East were recruited from one large clinic in Israel. Ophthalmologic investigations comprised of visual field assessments, intraocular pressure measurements, optic disc evaluation, and gonioscopy. Medical charts were obtained to date the onset of glaucoma and to evaluate aggressivity of the trait. We screened the myocilin gene (*MYOC*, OMIM 601652) by direct genomic sequencing of its three exons in all family members.

**Results:**

JOAG segregated as an autosomal dominant trait in four members of the family. The proband, a 14-year-old girl, had been diagnosed with juvenile open-angle glaucoma at 12 years old. Her mother, maternal aunt, and maternal grandfather all had JOAG that started at an early age. The disorder progressed rapidly even under optimal medical treatment, and all four patients had to undergo trabeculectomy. One missense mutation, Y371D (1111t→g, Tyr [Y] 371 Asp [D]), was identified. This mutation cosegregated with the disorder in all affected members and was absent in 200 Caucasian controls. The Y371D *MYOC* mutation has not been reported before. One cousin of the proband was a silent heterozygotic carrier of the mutation and was still asymptomatic at nine years of age.

**Conclusions:**

We identified a novel mutation (Y371D) in *MYOC* from a Caucasian family who presented with an aggressive form of JOAG that required early trabeculectomy. Genetic screening of the *MYOC* mutation was beneficial in predicting one asymptomatic heterozygotic carrier.

## Introduction

Open-angle glaucoma (OAG) is the most frequent form of glaucoma, accounting for more than half of all cases [1]. The increased frequency of OAG among relatives of patients with this condition indicates that its susceptibility is influenced by genetic factors. Ocular hypertension (OHT) above 21 mmHg is considered a major risk factor for OAG. According to the age of onset and aggressivity, OAG is divided into juvenile-onset OAG (JOAG) and adult-onset OAG. When associated with OHT, this form of OAG is known as primary open-angle glaucoma (POAG). JOAG has an earlier age of onset, between 10 and 35 years of age, and usually presents with high intraocular pressure (IOP), visual field loss, and optic disc damage. JOAG often requires early surgical treatment [2].

JOAG is typically inherited as an autosomal dominant trait whereas adult-onset POAG is considered a complex genetic trait [3,4]. More than 20 genetic loci have been mapped for POAG [3,4]. Among these, 14 genetic loci, designated GLC1A to GLC1N, have been defined for JOAG and/or POAG in family-based linkage studies of several pedigrees [3-10].

Five loci contribute to JOAG while the others exclusively account for adult-onset POAG [3,4]. Three of these five loci, 1q21-q31 (myocilin gene [*MYOC*], GLC1A), 9q22, and 20p12, have been confirmed in several studies [3,4]. Two additional studies have identified one novel locus on chromosome 15q22-q24 in one JOAG family and another on chromosome 5q in a different JOAG family [11,12].

Myocilin (*MYOC*) at GLC1A has been established as a direct cause of glaucoma [13]. In spite of intensive investigation, its function remains unknown. *MYOC* is primarily mutated in patients with JOAG [13,14]. Approximately 10%–20% of all JOAG cases are caused by mutations in *MYOC* [15].

*MYOC* consists of three exons with lengths of 604, 126, and 782 base pairs (bp) and encodes a 504 amino acid polypeptide [16]. Among 73 reported *MYOC* mutations, 63 (86.3%) are located in exon 3 (Myocilin allele-specific phenotype database), suggesting that the olfactomedin-like domain is important for POAG pathogenesis [17,18]. More than 50% of these mutations cause early onset severe glaucoma (JOAG) whereas a few cause late-onset POAG or even normal tension glaucoma (NTG) [19]. In Caucasians, mutations in *MYOC* account for as many as 36% of the families with JOAG but only for 2%–4% of sporadic patients with POAG [20,21]. We herein report a novel *MYOC* missense mutation in a Caucasian family that causes a characteristic JOAG phenotype spanning three generations.

## Methods

We investigated six members of a family originating from Uzbekistan and now living in the Middle East. When a patient has been diagnosed before moving to the Middle East, we obtained their ocular examination charts from the previous hospitals or clinics where they were first investigated.

Mutation analysis of *MYOC* was performed at the Laboratory of Ocular Genetics and Genomics (Québec City, Canada). Genomic DNA was extracted using the Puregene DNA isolation protocol (QIAGEN, Mississauga, Ontario, Canada) from whole blood drawn by venipuncture. Three *MYOC* amplicons were obtained by polymerase chain reaction (PCR) using the primer pairs described in a previously published study [21]. The myocilin genes were screened for sequence alterations by PCR and direct sequencing as previously reported [22] using an Applied Biosystems Prism 3730xl DNA Analyzer automated sequencer (Applied Biosystems Inc, Foster City, CA). Sequence data were analyzed using the Staden preGap4 and Gap4 programs [23]. Each familial proband was screened for mutations in all three exons of *MYOC*. Sequencing of the mutation was performed on both DNA strands.

## Results

### Phenotypic studies

A 14-year-old girl presented with progressive primary open-angle glaucoma in our glaucoma clinic two years ago when she was 12 years old. She was diagnosed at age 10 and treated with maximal topical therapy. Her visual acuity was then 20/20 in both eyes. Intraocular pressure was 24 and 28 mmHg in the right and left eyes, respectively. The anterior segment was normal on slit-lamp examination, and the angle was wide open on gonioscopy. The cup to disc ratio was 0.7 and 0.8 in the right and left eyes, respectively. Visual field testing revealed damage to both eyes. The left eye had upper and lower arcuate scotomas, and the right eye had superior arcuate scotoma and inferior nasal step. Trabeculectomy with mitomycin C and 5-fluorouracil was performed in her left eye at the age of 12 years, and then three months later, the procedure was performed in her right eye. She had refractive hypotony after both procedures and needed a second procedure for revision of the filter in both eyes. Today, at 14 years old, her visual acuity is 20/30 in the right eye and 20/20 in the left eye. Intraocular pressure (IOP) is 7 mmHg in the right eye and 10 mmHg in the left. She has large and raised filtering blebs. The cup to disc ratio has improved to 0.5 in both eyes, and there is also an improvement in the visual fields (this may seem quite unusual but possible in children) with lower nasal arcuate scotoma in the right eye and inferior nasal step scotoma in the left eye.

Her mother who is 32 years old, the mother’s sister who is 36 years old, and the father of these two women also suffer from progressive open-angle glaucoma. None of the patients had any other ocular or systemic abnormalities. The aunt of the proband, subject II-2, was recently admitted to our department as an emergency with end stage glaucoma in her only seeing (right) eye. Her visual acuity was 20/40, and the IOP was 50 mmHg. She was known to have juvenile glaucoma since the age of 16 in both eyes and was treated with topical and systemic medications to lower intraocular pressures. She became blind in her left eye after glaucoma surgery 12 years ago at the age of 24. She recently underwent uncomplicated trabeculectomy in her right eye, and today her visual acuity is 20/40 and intraocular pressure is 7 mmHg with a large diffuse filtering bleb and nearly total cup to disc ratio. Her visual field in her right eye was severely affected with only the central 10° remaining (tubular vision). The proband’s maternal aunt daugther (subject III-2), who is a nine-year-old, is currently healthy (Table 1).

**Table 1:** Phenotypic status of Israeli family members affected by OAG.

**Subject Number**	**Family member**	**Age today**	**Age at onset**	**Highest IOP OD/OS**	**Gonioscopy**	**VF–24–2 2009**	**MD OD/OS**	**PSD OD/OS**	**VA-2009**	**CD ratio OD/OS 2009**	**IOP today OD/OS 2009**	**Age of surgery: OD/OS**
I-1	grandfather	55	16	39/40	no data	No data	No data	No data	No data	No data	No data	No surgery
I-2	grandmother	61	-	22/22	no data	No data	No data	No data	No data	No data	No data	-
II-1	mother	32	18	No data	open	-	-	-	NLP/NLP	1/1	No data	No surgery
II-2	aunt	36	16	50/50	open	OD- tubular vision 10 °	No parameters (stimulus V)	No parameters (stimulus V)	OD-20/40 OS-NLP	0.9/1	10/14	36/24
III-1	proband	14	10	24/28	open	OD-lower arcuate scotoma OS- inferior nasal step	−7.18/-7.91	4/6.52 p<0.5%	OD-20/30 OS-20/20	0.5/0.7	11/12	12/12
III-2	cousin	9	Still asymptomatic	16/16 Feb 2007- the date of the last exam	open	No data	No data	No data	No data	normal	No data	No surgery

OD: right eye, OS: left eye. IOP: intraocular pressures, Age at onset in years, VF: visual fields, MD- mean deviation, PSD: pattern standard deviation, VA: visual acuity, CD: cup to disc ratio, IOP: intraocular pressure.

The cousin was found to be a heterozygotic carrier, and she is currently healthy at nine years of age. She will be followed up closely and treated if necessary. We were unable to examine additional family members as the family had lost contact with the husbands of the sisters and immigrated to Israel alone.

### Genotypic studies

**Figure 1:**
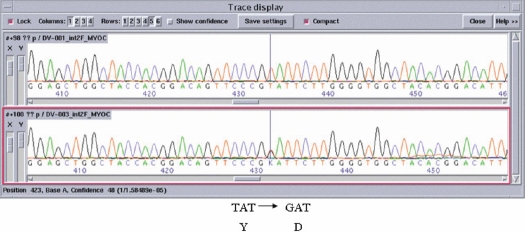
Sequence of the region of the mutation. *MYOC* sequence electropherogram  is shown of a wild-type unaffected subject (top panel) and of a heterozygous patient carrying the myocilin mutation, Y371D (lower panel). Nucleotides and predicted amino acid changes are indicated under the electropherogram. The vertical line points to the Y371D mutation.

**Figure 2:**
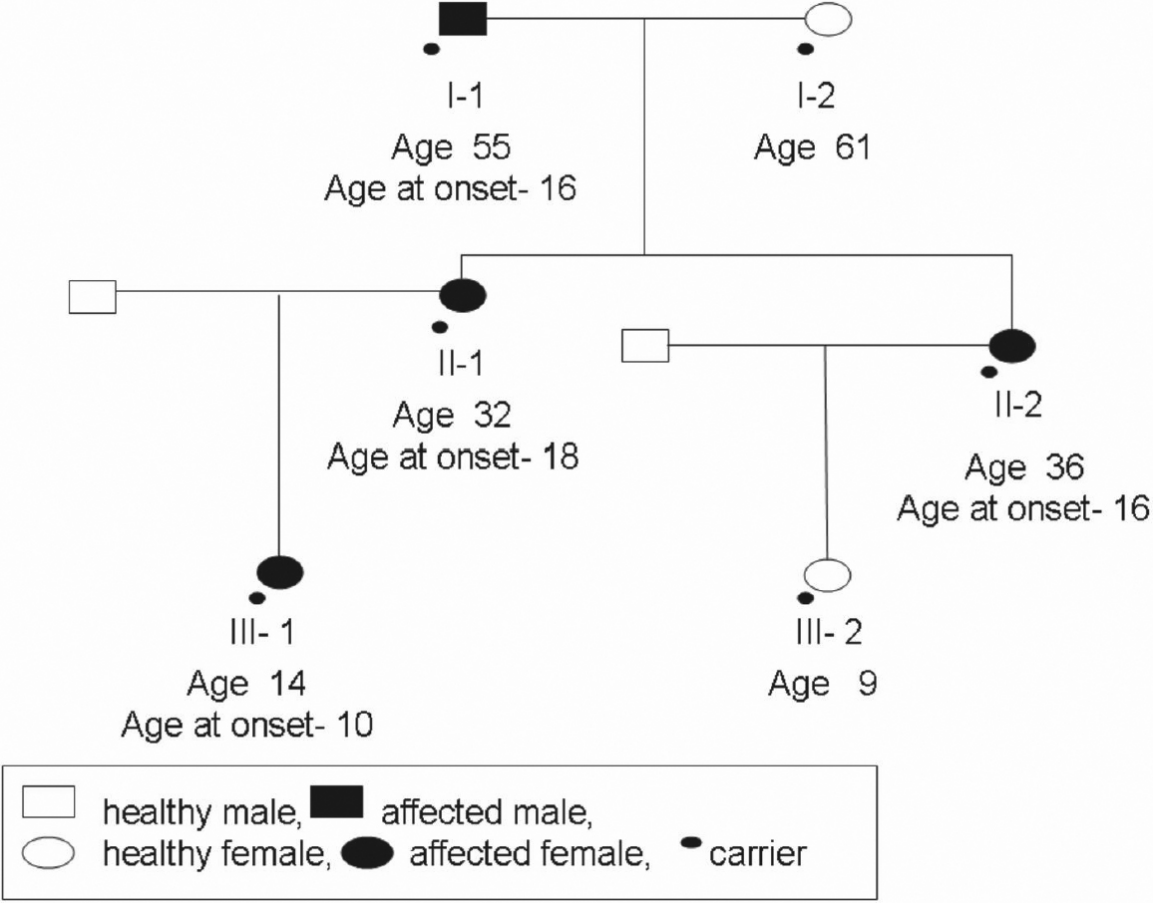
Segregation of the Y371D glaucoma-causing *MYOC* mutation in an Israeli pedigree. The phenotypic status of each subject is as described in the box and corresponds to Table 1. Heterozygotic carriers of the mutation are depicted by a small black dot under their own respective sign.

After screening all three exons of *MYOC*, a single T to G transition in exon 3 at position 1111t→g was detected in the coding sequence of *MYOC* (see Figure 1, which compares the normal and the mutated DNA sequences by ABI tracing). This transition changes the amino acid at position 371. As this transition was absent in more than 200 control persons (400 chromosomes) coming from all parts of the world and as this variation cosegregated with the disorder within the family, this Y371D change represents a mutation in exon 3. This mutation is novel since it has not been reported before as far as we know (see Myocilin allele-specific phenotype database). Figure 2 shows the segregation of the mutation in the family. This mutation causes autosomal dominant glaucoma.

## Discussion

We report a novel mutation (Y371D) in *MYOC* from a Caucasian family who presented with progressive open-angle glaucoma requiring early trabeculectomy. Specific mutations have been described in different population groups [24-29]. Cys433Arg, which is thought to be the most prevalent mutation in the Brazilian population, is associated with higher IOP and greater vertical cup/disc ratio when compared to patients without this mutation [28]. In Caucasian populations originating from Europe, the most frequently identified *MYOC* mutation is Gln368STOP, which has been reported in 1.65% of probands with POAG and has been associated with older-onset POAG and a lower level of IOP elevation [19,22,30].

As investigations of the molecular causes of glaucoma are now being undertaken in populations living in the Middle-East, it is envisaged that mutations not yet reported will be discovered in disease-causing genes. In this regard, the Y371D *MYOC* mutation is novel as far as we know. Interestingly, since this mutation was observed in a Caucasian family originating from Uzbekistan, it should be observed in other regions of the world.

Determining the clinical characteristics associated with particular *MYOC* mutations are essential to establish good prognosis and to initiate the most appropriate therapy. Phenotype/genotype correlation studies clearly established that patients carrying the *MYOC* Gly246Arg, Pro370Leu, or Tyr437His mutation displayed a severe clinical presentation appearing at a young age in children or in teenagers whereas those harboring the Gln368Stop mutation show a mild clinical presentation appearing at middle age or old age [24]. On the other hand, a few *MYOC* mutations exhibit variable expressivity of the phenotype. For instance, Wirtz et al. [31] described a family with an intermediate phenotype between juvenile and adult onset glaucoma with a *MYOC* Asp380His mutation while Morissette et al [32]. reported that the phenotypes associated with the *MYOC* Lys423Glu ranged from juvenile-onset to adult-onset open-angle glaucoma and showed either aggressive or mild phenotypes.

We present a family with open-angle glaucoma, which progressed aggressively. The aggressiveness of the glaucoma led us to perform filtering surgery in two members of this family (proband and her aunt) in a relatively short period of follow-up and at a younger age compared with most cases of open-angle glaucomas. The mother of the proband was already blind from glaucoma when first examined in our clinic.

The primary mechanism by which the *MYOC* Y371D mutation causes glaucoma may involve misfolding and intracellular sequestration of the mutant protein within the trabecular meshwork cell, thereby altering cell-mediated processes that control aqueous humor outflow. Indeed, several studies demonstrated that mutations occurring within the vicinity of amino acid 371 impeded secretion of heterodimers and multimers made of mutant myocilin polypeptides from interacting with their wild-type counterpart [33,34]. In particular, transfection experiments using COS-7 and human trabecular meshwork cells showed that myocilin mutant proteins, G364V, G367R, P370L, and D380A, were not secreted and remained within the intracellular milieu when studied at 37 °C [33,35].

Our phenotype/genotype correlation study on five patients clearly demonstrated here that the Y371D *MYOC* mutation caused juvenile onset glaucoma with a characteristic phenotype that includes onset in the second decade of life, usually high intraocular pressures, and a rapidly progressive OAG disease. We also described a sixth potential patient, a nine year-old carrier who is still asymptomatic. The gain of this report is in predicting the high probability that this asymptomatic cousin will become affected as the mutation is fully (100%) penetrant. Finally, observation of the reported mutation when screening for myocilin variations should help in managing other patients and their families treated for progressive open-angle glaucoma. This report emphasizes the importance of taking a good family history when investigating new glaucoma patients.
